# Skeletal Infections Caused by *Coccidioides* Species

**DOI:** 10.3390/diagnostics12030714

**Published:** 2022-03-15

**Authors:** Christos Koutserimpas, Symeon Naoum, Konstantinos Raptis, Georgia Vrioni, George Samonis, Kalliopi Alpantaki

**Affiliations:** 1 Department of Orthopaedics and Traumatology, “251” Hellenic Air Force General Hospital of Athens, 11525 Athens, Greece; chrisku91@hotmail.com (C.K.); naoumsimeon@gmail.com (S.N.); kraptis1981@hotmail.gr (K.R.); 2 Department of Microbiology, Medical School, National and Kapodistrian University of Athens, 11527 Athens, Greece; gvrioni@med.uoa.gr; 3 Department of Internal Medicine, University of Crete, 71500 Heraklion, Greece; 4 Department of Orthopaedics and Traumatology, “Venizeleion” General Hospital of Crete, 71409 Heraklion, Greece; apopaki@yahoo.gr

**Keywords:** fungal osteomyelitis, fungal spondylodiscitis, fungal osseous infection, *Coccidioides immitis*, *Coccidioides posadasii*, coccidioidomycosis

## Abstract

Background: Coccidioidomycosis represents an endemic and challenging disease, with rare extrapulmonary manifestations. The present review of all published cases of core and extremities osseous coccidioidomycosis aims to describe epidemiology, patients’ characteristics, symptoms as well as medical and surgical treatment options and their effectiveness. Methods: A thorough review of all published skeletal core and extremity infections due to *Coccidioides* species was conducted. Information regarding demographics, causative fungus, antifungal treatment (AFT), surgical management as well as the infection outcome was recorded. Results: A total of 92 cases of *Coccidioides* spp. skeletal infections were recorded in 87 patients. The patients’ mean age was 35.3 years. The most common site of infection was the spine (82.6%), followed by the foot (6.5%), while the predominant symptom upon presentation was pain (29.9%). Immunosuppressive conditions and/or medications were observed in 21 patients (24.1%). Regarding imaging methods, indicating diagnosis, plain X-rays or CT scans were performed in most patients (50.6%), followed by magnetic resonance imaging (MRI) (47.1%). Most cases were diagnosed through histopathology (62; 71.3%), followed by serology testing (36; 42.4%) and by cultures (35; 40.2%). In 32 cases (36.8%), *Coccidioides immitis* was cultured, while in the remaining 55 cases (63.2%) the fungus was not further characterized. Regarding AFT, amphotericin B was the preferred agent (52.9%), followed by fluconazole (43.3%). In most cases (78.2%) surgical treatment was also performed. Treatment was successful in 80.5% of cases. Conclusions: Skeletal core and extremity infections due to *Coccidioides* spp. represent a severe disease. With the available data, the combination of prolonged proper AFT with surgical intervention seems to be the optimal current therapeutic approach.

## 1. Introduction

Coccidioidomycosis represents a challenging clinical entity, further complicated with broad-range clinical manifestations [1]. Diagnosis is established through cultures and/or histopathology, while immunologic assays may also confirm the disease. Clinical infection ranges from asymptomatic to diverse manifestations, including pneumonia, as well as extrapulmonary disease, such as soft tissue and osteoarticular infections [2].

*Coccidioides* species, including *Coccidioides immitis* and *Coccidioides posadasii*, cause mainly pulmonary infections, known as “valley fever”, and are endemic to Southwestern United States. These fungi endure well in regions of low rainfall, few winter freezes and alkaline soil in Arizona, New Mexico, West Texas, the San Joaquin Valley of California and parts of Mexico and South America [1,2]. The disease was first described in Argentina and subsequently in California. The disease is primarily a pneumonic illness often confused with community-acquired pneumonia. Inhalation of aerosolized arthrospores represents the main route of infection, while direct inoculation is extremely rare. Symptomatic patients typically present as community-acquired pneumonia with fever, rash, headache, night sweats, arthralgia and myalgia. Pulmonary coccidioidomycosis is often mistaken for bacterial community-acquired pneumonia [2]. Clinical examination in combination with a meticulous medical history is essential to determine the proper diagnosis, while no pathognomonic findings in chest X-rays indicating an infection with *C. immitis* exist [1,3].

The disease has shown to exhibit seasonality as reported from California and Arizona. In California, the highest incidence occurs in the fall. It is of note that there was a large increase in cases in the Southern San Joaquin Valley from 1992 to 1995. The reported cases declined subsequently but never to pre-1992 numbers. In the last decade, there has been a steady increase in reported cases year over year [1,2,3].

In rare cases, approximately 1% of these infections, the skin, the musculoskeletal system and/or the meninges are affected [2]. It is also of note that many hosts exposed to the fungi via inhalation of aerosolized spores remain asymptomatic or have only mild respiratory symptoms that resolve without antifungal treatment [1,2].

Extrapulmonary infection occurs through hematogenous or lymphatic spread in the majority of cases, while it may disseminate to single or multiple sites [1]. In cases of musculoskeletal involvement, osteomyelitis represents the most common disease, while multiple sites may be affected. Furthermore, a strong predilection for the axial skeleton exists [3].

Spine involvement may range from discitis and paravertebral soft tissue infection to vertebral body erosion and neural compression. Extrapulmonary disseminated coccidioidomycosis with bone involvement is usually treated with proper antifungal agents and/or surgical treatment [4]. The initial therapy is most commonly fluconazole or itraconazole, with preference for itraconazole in bone and articular disease [1].

The present study is a thorough review of all published cases of core and extremities skeletal coccidioidomycosis in an effort to describe epidemiology, patients’ characteristics, symptoms as well as medical and surgical treatment options and their effectiveness.

## 2. Methods

A meticulous electronic search of the PubMed and MEDLINE databases was performed to identify all existing articles regarding cases of coccidioidal osteomyelitis of the upper and lower extremities and/or coccidioidal spondylodiscitis. The study period was from January 2000 to January 2022. Alone and in combination, the terms “*Coccidioides* osteomyelitis”, “*Coccidioides* spondylodiscitis”, “*Coccidioides* vertebral infection”, “coccidioidal osteomyelitis”, “fungal osteomyelitis”, “coccidioidal spondylitis”, “coccidioidal immitis spondylodiscitis”, “vertebral coccidioidomycosis”, “osseous coccidioidomycosis”, “musculoskeletal coccidioidomycosis”, “coccidioidal posadasii osteomyelitis” and “coccidioidal posadasii spondylodiscitis” were searched. Following the identification of these cases, individual references listed in each publication were further investigated for ascertainment of additional cases.

The present review was limited to papers published in English and in peer-reviewed journals. Expert opinions, book chapters; studies on animals or cadavers; in-vitro investigations as well as abstracts in scientific meetings were excluded. Additionally, cases of skeletal coccidioidomycosis in other sites apart from core and extremities (e.g., skull), as well as cases not including information regarding the antifungal treatment, were excluded. Furthermore, this review does not cover septic arthritis or prosthetic joint infection cases. 

The data extracted from these studies included age, gender, location of the infection, the presence of immunosuppressive condition and/or medications, symptomatology, duration and type of antifungal treatment (AFT), type of surgical intervention and imaging studies performed for the diagnosis. Furthermore, the results of medical and surgical treatment, along with the follow-up of each case, were recorded and evaluated. 

Treatment was considered successful if all signs and symptoms of the infection had disappeared and no recurrence was observed during the follow-up period. 

Data were recorded and analyzed using Microsoft Excel 2019 (Microsoft Corporation, Redmond, WA, USA).

## 3. Results

A total of 92 cases in 87 patients (69; 79.3% males), suffering skeletal infections caused by *Coccidioides* spp., covering a 21-year period, were identified [3,4,5,6,7,8,9,10,11,12,13,14,15,16,17,18,19,20,21,22,23,24,25,26]. The studied population’s mean age was 35.3 years (standard deviation (SD) = 15.0). (Table 1) Most cases were reported from the USA, while only two originated in other countries (case 20 in Japan and 31 in France, from Table 1). 

The most commonly affected site was the spine in 76 cases (82.6%), followed by the foot in 6 (6.5%), the patella and the hand in 3 each (3.3%), the knee in 2 (2.2%) and the pelvis and the rib cage in 1 each (1.1%). In two patients more than one site was affected (cases 23 and 26 in Table 1). 

In particular, as far as spine infections are concerned, the thoracic spine was the most commonly affected region (21 cases; 27.6%), followed by the lumbar (15; 19.7%) and the cervical spine (9; 11.8%), while the sacral and iliac spine were affected in 1 case each (1.3%). In 39 cases (42.4%), spine infection by *Coccidioides* spp. was reported, without mentioning the exact spine region.

As far coinfections with other microorganisms are concerned, two cases are reported. In particular, in case no. 33, coagulase-negative *Staphylococcus* had been also isolated, while in case no. 84, *Staphylococcus aureus*, *Klebsiella pneumonia* and *Enterobacter cloacae* had been also cultured.

Detailed information regarding immunosuppressive conditions, as well as symptomatology, is presented in Table 1. Moreover, 21 patients (24.1%) were immunocompromised, according to the available information from each report. Most of them (10; 47.6%) were commenced on corticosteroids, while five (23.8%) suffered diabetes mellitus. 

The predominant symptom upon presentation was pain, observed in 26 patients (29.9%), followed by local signs of infection, such as swelling and high local temperature in 10 (11.5%), while pyrexia and neurological deficits were present in six each (6.7%). 

Table 2 highlights diagnostic techniques, including imaging indicating the infection, as well as the methods of firm diagnosis of the fungus. Regarding imaging methods, plain X-rays or CT scans were performed in 44 patients (50.6%), followed by magnetic resonance imaging (MRI) in 41 (47.1%) and bone scans in 8 (9.2%). In 39 cases (44.8%) the exact imaging study conducted was not reported.

Definite diagnosis was possible through cultures, histopathology and/or serology tests. Moreover, in 15 cases (17.2%), *Coccidioides immitis* was cultured, while in the remaining 72 cases (82.8%) the fungus was not further characterized (*Coccidioides* spp.). In particular, most cases of *Coccidioides* spp. skeletal infections were diagnosed through histopathology (62; 71.3%), followed by serology testing (36; 42.4%) and by cultures (35; 40.2%). Both histopathology and cultures were positive for *Coccidioides* in 22 cases (25.3%), cultures and serology tests in 21 (24.1%), while all three diagnostic methods were positive in 15 cases (17.2%).

Table 3 highlights the management of the reported cases, as well as outcome of the infection. Regarding AFT, 44 cases (50.6%) were treated with a single antifungal regimen; 32 (36.8%) with two, either simultaneously or consecutively, while 11 (12.6%) were treated with more than two antifungal regimens. Information regarding the duration of AFT was not reported in 60 cases (70%). The mean duration of treatment was found to be 19.8 months (SD = 18.1).

Amphotericin B was the preferred agent in 46 cases (52.9%, in 11 (23.9%) as monotherapy), followed by fluconazole in 38 cases (43.3%, in 28 (73.7%) as monotherapy), itraconazole in 17 (19.5%, in 5 (29.4%) as monotherapy), voriconazole in 8 (9.2%, none as monotherapy), ketoconazole in 3 (3.4%, in 1 (1.1%) as monotherapy), caspofungin in 1 (2.3%, not as monotherapy) as well as posaconazole in 1 (1.1%, not as monotherapy).

During the 2000–2022 period, the outcome was successful in 70 cases (80.5%), while mortality rate was 2.3%. Infection outcome was not reported (unknown) in three cases. 

A total of 68 patients (78.2%) underwent surgery for infection management. The outcome in patients receiving surgical and AFT was successful in 56 cases (86.2%), while the outcome in patients receiving only AFT was successful in 13 cases (59.1%).

## 4. Discussion

Coccidioidomycosis, also known as “valley fever” or “desert rheumatism”, represents a fungal infection caused by the soil-dwelling fungi, *C. immitis* and *C. posadasii*, which are found in various endemic places [27,28]. Infection is typically caused by inhalation, while hosts usually remain asymptomatic. However, the disease sometimes presents as pneumonia that may be either mild or, on certain occasions, life-threatening with dissemination in other sites of the body, such as the skin, bones and/or joints and meninges [29].

It is estimated that about 70% of all coccidioidal infections in United States occur in Arizona and 25% in California. Moreover, 150,000 new cases per year are estimated to occur in Southwestern US, while the annual incidence of coccidioidomycosis is constantly increasing [27,30]. It is believed that the infection is also present in Latin America and Northwest Mexico, where climatic conditions are similar to Arizona and California. However, the present review did not locate any skeletal coccidioidomycosis cases originating in these countries. The great rise in coccidioidal infections, especially in endemic areas, has been attributed to numerous factors, such as growing population, migration of people to endemic places, increased number of the elderly or individuals with immunosuppression/medical conditions exposed to soil disturbances due to construction and businesses as well as climate alterations [30]. It must be noted that since the whole world has become a global village, with easy communication and travelling, it cannot be excluded that coccidioidomycosis cases may appear in non-endemic areas, such as Europe and other regions where the infection is rare. Hence, symptomatology and treatment of the disease should be known to infectious disease physicians all over the world. The present review represents an opportunity to educate health practitioners who are not familiar with this infection in non-endemic areas.

Furthermore, the public health impact of coccidioidomycosis is notable, since approximately 25,000 hospitalizations due to this infection or to its complications as well as approximately USD 2 billion in hospital charges are recorded in the state of California during the period 2000–2011 [29]. It is of note that dust moving by air currents may be dangerous if inhaled, since it may contain *Coccidioides* aerosolized arthrospores. Additionally, dust originating in construction works may contain various molds, such as *Aspergillus* and *Coccidioides*. Hence, the use of protective masks in endemic areas as well as wetting of soil originating in construction represent a necessity for people exposed to such conditions [31].

Dissemination of the infection beyond the respiratory system occurs in a small percentage of cases, probably in less than 1% of all coccidioidomycosis cases, with the accurate incidence still being unclear [32]. Male gender, pregnancy, ethnicity (African or Filipino ancestry) and suppression of the cellular immunity, such as HIV infection, organ transplantation and chronic immunosuppressive medication, have been documented as risk factors for disseminated coccidioidomycosis infection [33]. Natural disasters have been documented to affect the environmental release of fungal spores [1,2]. Coccidioidomycosis has also been associated with a few instances of post-disaster infection [1,2].

The present study reviewed all published cases of skeletal infections caused by *Coccidioides* spp. during the period 2000–2022 in an attempt to clarify epidemiology, patients’ characteristics, symptomatology, medical and surgical treatment options as well as the infection outcome. This study reviewed 92 cases in 87 patients with osseous *Coccidioides* spp. infections during a 21-year period. The studied population was rather young (mean age = 35.3 years), while the male gender was highly represented (males = 79.3%). It is of note that most patients of the present study did not suffer impairment of the cell-mediated immunity, since they did not suffer any underlying immunosuppressive condition, and they were not receiving medications that could increase the possibility of complications. Additionally, immunosuppression has great impact on the clinical severity of coccidioidomycosis by magnifying the risk of disseminated infection [34,35]. In the present review, 24.1% of the studied population were immunocompromised, according to the available information from each report. 

The musculoskeletal system is a quite common site affected in disseminated disease, while osseous involvement appears in approximately 10 to 50% of disseminated infection cases [33]. In such cases, it has been documented by a recent study that 18.7% of patients exhibited involvement of the core (axial skeleton) and 17.3% of the extremities (appendicular skeleton). As far as the axial skeleton is concerned, vertebral lesions were almost always present. Furthermore, of the patients having osteoarticular infections, 78.6% had radiographic findings of related osteomyelitis [33]. In the present study, the spine was the most commonly affected region (82.6%), followed by foot (6.5%), patella (3.3%), hand (3.3%), knee (2.2%), pelvis and ribs (1.1% each). In particular, regarding spinal infections, the thoracic spine was the most commonly affected region (27.6%), followed by the lumbar (19.7%), the cervical (11.8%), the sacral and the iliac spine (1.3% each). Additionally, infection in multiple sites has also been reported [33]. In this study, in two patients more than one site was affected (2.3%).

Onset of fungal infections is usually devious with general symptoms and, as a result, diagnosis may be delayed and challenging [36,37]. In the present study, the predominant symptom of fungal skeletal infection caused by *Coccidioides* spp. was pain (29.9%), followed by local inflammation signs (11.5%), pyrexia and neurological symptomatology (6.7% each). Pain, tenderness, swelling and impaired range of motion are common clinical features not only for fungal but for other infectious skeletal diseases [37]. In addition, no reliable differentiation between bacterial and skeletal coccidioidomycosis is available, due to lack of any particular clinical manifestations [2,36]. Consequently, the combination of both clinical and imaging findings of a probable osseous infection makes laboratory diagnosis of paramount importance so that the responsible microorganism may be isolated and identified. 

Two *Coccidioides* species, *C. immitis* and *C. posadasii*, are responsible for fungal infections in humans. In the present review, *C. immitis* was isolated in 15 cases (17.2%), while in the remaining 72 cases (82.8%) the fungus was not further characterized. In fungal culture, *Coccidioides* species can be identified, while *C. immitis* species identification requires PCR and genomic analysis. These methods are still not widely available in everyday clinical practice. The two species have differences regarding their genomes. However, no variation in disease development, diagnosis or treatment has been reported. In fact, pathogenicity of the two species has been suggested to be quite similar [29,38]. It is of note that in only two cases mixed infections with bacteria were reported. 

In all the studied cases, the causative species had been isolated and identified through histopathology, cultures and/or through serology. In particular, the majority of cases were diagnosed through histopathological examination (71.3%), followed by serology testing (42.4%) and cultures (40.2%). Both histopathology and cultures were positive for *Coccidioides* in 25.3% of patients, cultures and serology tests in 24.1%, while all three diagnostic methods were positive in 17.2% of patients. Regarding imaging studies, more than half of patients (50.6%) had abnormal suggestive findings for skeletal infection in radiographs or CT scans, followed by MRI (47.1%) and bone scans (9.2%). Despite the fact that numerous laboratory tests and imaging studies could be abnormal in patients with coccidioidal infection, these results are non-specific [39]. Most patients suspected for coccidioidomycosis are examined through serology. Nevertheless, there are certain restrictions, as there may be delay in the development of serum antibodies from the infection onset, lasting several weeks, especially in immunocompromised patients [40,41]. Hence, coccidioidomycosis should not be excluded from the differential diagnosis, solely due to the absence of detectable anti-coccidioidal antibodies, especially during the early phase of the disease. 

Furthermore, isolating and locating the microorganism in respiratory secretions, tissue or other body specimens by either identifying the fungus in histological examination or by growing it from fungal cultures is definitive evidence of a coccidioidal infection. As far as staining characteristics are concerned, spherules are the most regular morphologic form of *Coccidioides* observed in human specimens [42]. The easiest means to distinguish spherules is to create a “wet preparation” using saline or potassium hydroxide solution. Calcofluor staining could also ameliorate direct detection of spherules. For tissue specimens, hematoxylin and eosin staining are usually adequate to recognize spherules. Sometimes, particular stains, such as periodic acid Schiff or Grocott or Gomori-methenamine silver are used for identification of the causative organisms [42]. Positive cultures may be the earliest and, on some occasions, a unique method of diagnosis. In order for the isolated fungus to be ascertained as belonging to *Coccidioides* spp., additional testing is usually required. This can be delivered through genetic probing detecting *Coccidioides*-specific DNA. This test is reported to be quite rapid and extremely trustworthy [43]. *C. immitis* and *C. posadasii* may not be distinguished through the commercially available tests [44]. In addition, an antigen method for *Coccidioides* spp. is available, and it may be detected either in urine sample or in blood of cases with disseminated infection [45]. Real-time polymerase chain reaction (RT-PCR) testing has been used for coccidioidomycosis diagnosis in a variety of clinical occasions [46,47]. The specificity of this method is reported to be considerably high, despite the fact that its sensitivity may not surpass that of culture. Furthermore, RT-PCR may be applied for formalin-fixed tissue, with faster results compared to culture methodology [46,48]. It should be noted that the RT-PCR method is available at an increasing number of specific laboratories [47,49].

Antifungal therapy is strongly recommended in all patients with osseous and joint coccidioidal infection [40]. Triazole agents, as well as amphotericin B, are the antifungals used for the treatment of coccidioidomycosis [29]. The findings of the present study have shown that about 50.6% of cases were treated with a single antifungal regimen, 36.8% with two, either simultaneously or consecutively, while 12.6% were treated with more than two antifungal regimens. 

Amphotericin B was the preferred antifungal regimen (52.9% of the studied cases), while in 23.9% it was used as monotherapy. Amphotericin B is an efficient broad spectrum antifungal agent. However, its relative toxicities, as well as its side effects, including renal dysfunction, are limiting its essential long-term application [50,51,52]. The liposomal compounds of amphotericin B have minimized this agent’s nephrotoxicity, but kidney function may still be impaired by the extended drug usage [53]. Information regarding the type of amphotericin B was not accessible in most cases; it is, nevertheless, presumed that lipid or liposomal compounds of amphotericin B have been the agents of choice due to their milder side effects, in comparison with deoxycholate amphotericin B, an agent that has been practically abandoned during the last 20 years. 

Fluconazole is reported to be the most widely used antifungal agent against coccidioidomycosis, due to its relatively low cost and availability in either intravenous or oral formulations [54]. This drug’s oral bioavailability is excellent, while there is no alteration by food or gastric diseases. Additionally, fluconazole is not protein bound and is allocated widely in the majority of human tissues and fluids [55,56]. In the present study, fluconazole was used in 43.3% of patients, mainly as a monotherapy (73.7%). Itraconazole is also widely used, and there is some evidence that its administration is superior regarding treatment of some disseminated coccidioidal infections [54,57]. For cases with severe osteoarticular disease, such as limb-threatening skeletal disease or vertebral infection causing imminent cord compromise, a combination of a triazole agent and amphotericin B is indicated. Based on clinical evidence, the combination of triazole and amphotericin B seems to accelerate recovery and simplify the transition to triazole therapy alone [58].

The major drug–drug interactions related to azole agents include oxidative drug metabolism via the cytochrome P450 (CYP) system. All triazoles are metabolized by and affect the P450 enzyme system to a varying extent [59,60]. Fluconazole is a strong inhibitor of CYP2C19 and a moderate inhibitor of CYP2C9 and CYP3A4. In general, this does not cause any concern for doses less than 200 mg per day [61]. It is also an inhibitor of uridine 5′-diphosphate glucuronosyltransferase (UGT) enzymes. Fluconazole is only a weak substrate of CYP450 enzymes. Voriconazole is a strong inhibitor of CYP3A4, a moderate inhibitor of CYP2C19 and a weak inhibitor of CYP2C9 isoenzymes. Voriconazole is metabolized extensively by CYP2C19 and CYP3A4 and, to a minor extent, by CYP2C9. Since CYP2C9 and CYP2C19 exhibit genetic polymorphism, wide variations in pharmacokinetics are noticed among specific populations [61].

Depending on the severity of skeletal coccidioidal infections, surgical intervention may be necessary. The majority of the reviewed cases (78.2%) underwent surgical intervention for the eradication of the infection. Vertebral instability, neurological deterioration/impairment as well as infection development while the patient had been already commenced on antifungal therapy should be carefully evaluated and thoroughly considered as criteria for potential surgical interference [53]. It is of note that AFT without surgery is usually quite efficient in entirely eliminating the signs and manifestations of the infection. However, surgical debridement or stabilization are highly recommended and should be considered when abscesses, spinal instability or impingement on an organ or tissue are evident. In the present review, the outcomes in patients receiving both surgical and AFT was successful in 86.2% of cases, while the outcome in patients receiving only AFT was successful in 59.1%. 

The present study has some limitations. Dosages, drug serum-levels, MICs and side effects of the used antifungal agents were not described in most cases. Furthermore, due to the heterogeneity of skeletal infections, including limb as well as vertebral infections, the type of surgery varies vastly. Hence, it cannot be categorized. However, in all patients undergoing surgery, surgical debridement was also performed. Nevertheless, this review provides valuable information about epidemiology, symptomatology, treatment as well as outcome of cases of skeletal infection caused by *Coccidioides* spp.

In conclusion, osseous core and extremity infections due to *Coccidioides* spp. represent a severe disease, requiring prompt definite diagnosis and multidisciplinary management. In most cases surgical intervention is necessary. The combination of prolonged proper AFT with surgical intervention seems to be the optimal current therapeutic approach. Furthermore, in cases of skeletal infections, especially in the southwest region of the United States, proven culture negative for bacteria and/or cocci, high index of suspicion for *Coccidioides* spp. should be present.

## Figures and Tables

**Table 1:** Patient demographics, responsible fungus, affected region Charlson Comorbidity Index, immunosuppressive condition/medical history and symptoms. M: male, F: female, LSI: local signs of inflammation.

Case No	Year	Author	Age/ Gender	*Coccidioides* Species	Affected Region	Immunosuppressive Conditions/Medical History	Symptoms
**1.**	2001	Wrobel et al. [5]	M/48	*Coccidioides* spp.	Spine (Thoracic)	-	-
**2.**	2001	Wrobel et al. [5]	F/62	*Coccidioides* spp.	Spine (Thoracic)	-	-
**3.**	2001	Wrobel et al. [5]	M/19	*Coccidioides* spp.	Spine (Lumbar)	-	-
**4.**	2001	Wrobel et al. [5]	M/34	*Coccidioides* spp.	Spine (Thoracic)	-	-
**5.**	2001	Wrobel et al. [5]	M/28	*Coccidioides* spp.	Spine (Thoracic and Lumbar)	-	-
**6.**	2001	Wrobel et al. [5]	M/21	*Coccidioides* spp.	Spine (Lumbar)	-	-
**7.**	2001	Wrobel et al. [5]	M/28	*Coccidioides* spp.	Spine (Thoracic)	-	-
**8.**	2001	Wrobel et al. [5]	M/53	*Coccidioides* spp.	Spine (Thoracic)	-	-
**9.**	2001	Wrobel et al. [5]	M/40	*Coccidioides* spp.	Spine (Thoracic)	-	-
**10.**	2001	Wrobel et al. [5]	M/23	*Coccidioides* spp.	Spine (Cervical)	-	-
**11.**	2001	Wrobel et al. [5]	F/26	*Coccidioides* spp.	Spine (Thoracic)	-	-
**12.**	2001	Wrobel et al. [5]	M/29	*Coccidioides* spp.	Spine (Thoracic and Lumbar)	-	-
**13.**	2001	Wrobel et al. [5]	M/42	*Coccidioides* spp.	Spine (Lumbar)	-	-
**14.**	2001	Wrobel et al. [5]	M/27	*Coccidioides* spp.	Spine (Thoracic and Lumbar)	-	-
**15.**	2001	Wrobel et al. [5]	F/13	*Coccidioides* spp.	Spine (Lumbar)	-	-
**16.**	2001	Wrobel et al. [5]	M/9	*Coccidioides* spp.	Spine (Lumbar)	-	-
**17.**	2001	Wrobel et al. [5]	M/34	*Coccidioides* spp.	Spine (Cervical and Thoracic)	-	-
**18.**	2001	Wrobel et al. [5]	M/39	*Coccidioides* spp.	Spine (Lumbar)	Sarcoidosis, corticosteroids	Pain
**19.**	2001	Wrobel et al. [5]	M/45	*Coccidioides* spp.	Spine (Lumbar)	-	-
**20.**	2002	Nakamura et al. [6]	Μ/28	*Coccidioides immitis*	Spine (Thoracic)	-	Pyrexia
**21.**	2003	Copeland et al. [7]	M/34	*Coccidioides immitis*	Spine (Cervical)	Alcohol and drug use	Pain, LSI
**22.**	2003	Kirk KL and Kuklo TR. [8]	M/22	*Coccidioides* spp.	Spine (Thoracic)	-	Pain, fatigue
**23.**	2004	Arnold et al. [9]	F/17	*Coccidioides immitis*	Foot (Metatarsal), Maxilla, Knee, Hands and Spine (Lumbar and Thoracic)	-	Pain
**24.**	2004	Arnold et al. [9]	M/29	*Coccidioides immitis*	Spine (Iliac)	-	Pain, LSI
**25.**	2004	Lewickyet al. [10]	F/36	*Coccidioides immitis*	Spine (Lumbar)	Sarcoidosis, corticosteroids	Pain, pyrexia, shortness of breath
**26.**	2004	Prabhu et al. [11]	M/31	*Coccidioides* spp.	Ribs, pelvis, spine (Cervical, Thoracic, Lumbar, Sacral)	-	Pain, pyrexia, LSI
**27.**	2005	Taxy JB and Kodros S [12]	M/22	*Coccidioides immitis*	Foot (ankle)	-	-
**28.**	2005	Taxy JB and Kodros S [12]	F/53	*Coccidioides immitis*	Spine (Thoracic)	-	Neurological symptomatology
**29.**	2006	Sandoval et al. [13]	M/39	*Coccidioides immitis*	Foot	Hepatitis C, liver failure	Pain, LSI
**30.**	2008	Sheppard JE and Switlick DN [14]	F/7	*Coccidioides immitis*	Radius	-	Pain, LSI
**31.**	2010	Reach et al. [15]	F/28	*Coccidioides immitis*	Spine (Thoracic and Lumbar)	-	Pain, pyrexia, weight loss
**32.**	2010	Waterman et al. [16]	M/11	*Coccidioides* spp.	Patella	-	Pain, intermittent locking.
**33.**	2011	Ho et al. [17]	M/50	*Coccidioides immitis*	Foot (fifth metacarpal)	Chronic hepatitis B and C	Pain, LSI
**34.**	2011	Kakarla et al. [18]	M/48	*Coccidioides* spp.	Spine (Cervical)	-	Pain, fatigue, pyrexia
**35.**	2011	Kakarla et al. [18]	M/49	*Coccidioides* spp.	Spine (Cervical)	-	Pain
**36.**	2011	Kakarla et al. [18]	M/50	*Coccidioides* spp.	Spine (Thoracic)	-	Pain, neurological symptomatology
**37.**	2012	El Abd et al. [19]	F/48	*Coccidioides* spp.	Spine (Thoracic)	Systemic lupus erythematosus, Sjögren syndrome, chronic fatigue syndrome, depression, insomnia, fibromyalgia, hypothyroidism, corticosteroids	Pain
**38.**	2012	Szeyko et al. [20]	M/17	*Coccidioides* spp.	Spine (NR)	-	-
**39.**	2012	Szeyko et al. [20]	M/17	*Coccidioides* spp.	Spine (NR)	-	-
**40.**	2012	Szeyko et al. [20]	M/19	*Coccidioides* spp.	Spine (NR)	-	-
**41.**	2012	Szeyko et al. [20]	M/20	*Coccidioides* spp.	Spine (NR)	-	-
**42.**	2012	Szeyko et al. [20]	M/20	*Coccidioides* spp.	Spine (NR)	-	-
**43.**	2012	Szeyko et al. [20]	M/21	*Coccidioides* spp.	Spine (NR)	-	-
**44.**	2012	Szeyko et al. [20]	M/21	*Coccidioides* spp.	Spine (NR)	Sarcoidosis, corticosteroids	-
**45.**	2012	Szeyko et al. [20]	M/22	*Coccidioides* spp.	Spine (NR)	-	-
**46.**	2012	Szeyko et al. [20]	M/22	*Coccidioides* spp.	Spine (NR)	-	-
**47.**	2012	Szeyko et al. [20]	M/25	*Coccidioides* spp.	Spine (NR)	-	-
**48.**	2012	Szeyko et al. [20]	M/27	*Coccidioides* spp.	Spine (NR)	-	-
**49.**	2012	Szeyko et al. [20]	M/31	*Coccidioides* spp.	Spine (NR)	-	-
**50.**	2012	Szeyko et al. [20]	M/34	*Coccidioides* spp.	Spine (NR)	-	-
**51.**	2012	Szeyko et al. [20]	M/36	*Coccidioides* spp.	Spine (NR)	HIV, corticosteroids	-
**52.**	2012	Szeyko et al. [20]	M/36	*Coccidioides* spp.	Spine (NR)	-	-
**53.**	2012	Szeyko et al. [20]	M/38	*Coccidioides* spp.	Spine (NR)	HIV	-
**54.**	2012	Szeyko et al. [20]	M/39	*Coccidioides* spp.	Spine (NR)	-	-
**55.**	2012	Szeyko et al. [20]	M/39	*Coccidioides* spp.	Spine (NR)	-	-
**56.**	2012	Szeyko et al. [20]	M/41	*Coccidioides* spp.	Spine (NR)	-	-
**57.**	2012	Szeyko et al. [20]	M/44	*Coccidioides* spp.	Spine (NR)	-	-
**58.**	2012	Szeyko et al. [20]	M/44	*Coccidioides* spp.	Spine (NR)	-	-
**59.**	2012	Szeyko et al. [20]	M/44	*Coccidioides* spp.	Spine (NR)	Sarcoidosis, corticosteroids	-
**60.**	2012	Szeyko et al. [20]	M/45	*Coccidioides* spp.	Spine (NR)	-	-
**61.**	2012	Szeyko et al. [20]	M/46	*Coccidioides* spp.	Spine (NR)	-	-
**62.**	2012	Szeyko et al. [20]	M/47	*Coccidioides* spp.	Spine (NR)	-	-
**63.**	2012	Szeyko et al. [20]	M/50	*Coccidioides* spp.	Spine (NR)	-	-
**64.**	2012	Szeyko et al. [20]	M/50	*Coccidioides* spp.	Spine (NR)	-	-
**65.**	2012	Szeyko et al. [20]	M/50	*Coccidioides* spp.	Spine (NR)	Diabetes mellitus	-
**66.**	2012	Szeyko et al. [20]	M/54	*Coccidioides* spp.	Spine (NR)	Diabetes mellitus	-
**67.**	2012	Szeyko et al. [20]	M/57	*Coccidioides* spp.	Spine (NR)	Corticosteroids	-
**68.**	2012	Szeyko et al. [20]	M/67	*Coccidioides* spp.	Spine (NR)	-	-
**69.**	2012	Szeyko et al. [20]	F/16	*Coccidioides* spp.	Spine (NR)	-	-
**70.**	2012	Szeyko et al. [20]	F/20	*Coccidioides* spp.	Spine (NR)	-	-
**71.**	2012	Szeyko et al. [20]	F/20	*Coccidioides* spp.	Spine (NR)	Systemic lupus erythematosus, corticosteroids	-
**72.**	2012	Szeyko et al. [20]	F/21	*Coccidioides* spp.	Spine (NR)	Corticosteroids	-
**73.**	2012	Szeyko et al. [20]	F/25	*Coccidioides* spp.	Spine (NR)	-	-
**74.**	2012	Szeyko et al. [20]	F/27	*Coccidioides* spp.	Spine (NR)	-	-
**75.**	2012	Szeyko et al. [20]	F/52	*Coccidioides* spp.	Spine (NR)	-	-
**76.**	2012	Szeyko et al. [20]	F/66	*Coccidioides* spp.	Spine (NR)	Diabetes mellitus	-
**77.**	2013	Zhu et al. [21]	M/65	*Coccidioides* spp.	Foot (Ankle)	Coronary artery disease, heart failure, hypertension, tobacco use	Pain
**78.**	2014	Berli et al. [22]	M/35	*Coccidioides immitis*	Hand (Ring Finger Metacarpal)	Tuberculosis	Pain, LSI
**79.**	2014	Li et al. [23]	M/27	*Coccidioides* spp.	Patella	-	Pain, limited range of motion
**80.**	2014	Li et al. [23]	M/78	*Coccidioides* spp.	Patella	-	Pain
**81.**	2014	Tan et al. [24]	M/55	*Coccidioides immitis*	Spine (Cervical)	Sarcoidosis, corticosteroids	Neurological symptomatology
**82.**	2015	Ellerbrook L and Laks S. [3]	M/21	*Coccidioides* spp.	Knee	Diabetes mellitus	Pain, LSI
**83.**	2017	Khalid et al. [25]	M/17	*Coccidioides immitis*	Foot (Right First Toe)	Diabetes mellitus	Pain, LSI
**84.**	2017	McConnell et al. [26]	M/33	*Coccidioides immitis*	Spine (Cervical and Thoracic)	-	Pain, LSI
**85.**	2019	Ramanathan et al. [4]	M/28	*Coccidioides* spp.	Spine (Thoracic)	-	Pain, shortness of breath, pyrexia
**86.**	2019	Ramanathan et al. [4]	M/62	*Coccidioides* spp.	Spine (Lumbar)	-	Pain, neurological symptomatology
**87.**	2019	Ramanathan et al. [4]	M/54	*Coccidioides* spp.	Spine (Cervical)	-	Pain, neurological symptomatology

**Table 2:** Definite diagnosis of skeletal infection caused by *Coccidioides* spp. and imaging techniques that each case underwent during the process of diagnosing the infection. MRI: magnetic resonance imaging, CT: computer tomography, NR: not reported (although the specimen (bone, tissue, abscess, etc.) studied is not reported, the technique is).

Case	MRI	C/T X-ray	Bone Scanning with ^99m^Tc	Cultures	Biopsy	Other
1.	+	+	-	-	tissue specimen	-
2.	+	+	-	-	tissue specimen	-
3.	+	+	-	-	tissue specimen	-
4.	+	+	-		tissue specimen	-
5.	+	+	-	-	tissue specimen	-
6.	+	+	-	-	tissue specimen	-
7.	+	+	-	-	tissue specimen	-
8.	+	+	-	-	tissue specimen	-
9.	+	+	-	-	tissue specimen	-
10.	+	+	-	-	tissue specimen	-
11.	+	+	-	-	tissue specimen	-
12.	+	+	-	-	tissue specimen	-
13.	+	+	-	-	tissue specimen	-
14.	+	+	-	-	tissue specimen	-
15.	+	+	-	-	tissue specimen	-
16.	+	+	-	-	tissue specimen	-
17.	+	+	-	-	tissue specimen	-
18.	+	+	+	-	tissue specimen	-
19.	+	+	-	-	tissue specimen	-
20.	+	+	-	drainage material	-	-
21.	-	+	-	drainage material	-	-
22.	+	+	+		bone specimen	serology
23.	-	-	+	tissue specimen		serology
24.	-	-	+	tissue specimen	-	serology
25.	-	+	+	-	tissue specimen	serology
26.	+	+	-	-	tissue specimen	-
27.	-	+	-	tissue specimen	tissue specimen	-
28.	-	+	-	tissue specimen	tissue specimen	-
29.	+	+	-	tissue specimen	-	-
30.	+	+	+	tissue specimen	tissue specimen	serology
31.	+	+	-	tissue specimen	tissue specimen	-
32.	+	+	-	tissue specimen	tissue specimen	-
33.	+	+	-	tissue specimen	-	-
34.	+	+	-	-	tissue specimen	-
35.	+	+	-	-	tissue specimen	-
36.	+	+	-	-	tissue specimen	-
37.	+	+	+	tissue specimen	-	-
38.	NR	NR	NR	-	-	serology
39.	NR	NR	NR	-	-	serology
40.	NR	NR	NR	-	-	serology
41.	NR	NR	NR	-	-	serology
42.	NR	NR	NR	tissue specimen	tissue specimen	serology
43.	NR	NR	NR	tissue specimen	-	-
44.	NR	NR	NR	-	tissue specimen	-
45.	NR	NR	NR	-	-	serology
46.	NR	NR	NR	tissue specimen	tissue specimen	serology
47.	NR	NR	NR	-	tissue specimen	-
48.	NR	NR	NR	tissue specimen	tissue specimen	serology
49.	NR	NR	NR	-	tissue specimen	serology
50.	NR	NR	NR	tissue specimen	tissue specimen	serology
51.	NR	NR	NR	-	tissue specimen	-
52.	NR	NR	NR	tissue specimen	tissue specimen	-
53.	NR	NR	NR	tissue specimen	tissue specimen	-
54.	NR	NR	NR	tissue specimen	-	serology
55.	NR	NR	NR	tissue specimen	tissue specimen	serology
56.	NR	NR	NR	-	tissue specimen	-
57.	NR	NR	NR	tissue specimen	tissue specimen	serology
58.	NR	NR	NR	-	-	serology
59.	NR	NR	NR	-	-	serology
60.	NR	NR	NR	tissue specimen	tissue specimen	serology
61.	NR	NR	NR	tissue specimen	tissue specimen	serology
62.	NR	NR	NR	tissue specimen	tissue specimen	serology
63.	NR	NR	NR	tissue specimen	tissue specimen	serology
64.	NR	NR	NR	-	-	serology
65.	NR	NR	NR	-	-	serology
66.	NR	NR	NR	-	tissue specimen	-
67.	NR	NR	NR	-	tissue specimen	-
68.	NR	NR	NR	tissue specimen	-	serology
69.	NR	NR	NR	tissue specimen	-	serology
70.	NR	NR	NR	-	-	serology
71.	NR	NR	NR	tissue specimen	tissue specimen	serology
72.	NR	NR	NR	tissue specimen	tissue specimen	serology
73.	NR	NR	NR	-	-	serology
74.	NR	NR	NR	-	tissue specimen	-
75.	NR	NR	NR	-	tissue specimen	-
76.	NR	NR	NR	-	tissue specimen	-
77.	+	-	-	-	tissue specimen	-
78.	+	+	-	tissue specimen	tissue specimen	-
79.	+	+	-		-	serology
80.	-	+	+	tissue specimen	tissue specimen	serology
81.	+	+	-	tissue specimen	tissue specimen	serology
82.	+	+	-	-	tissue specimen	-
83.	+	+	-	tissue specimen	-	serology
84.	+	+	-	tissue specimen	-	-
85.	+	+	-	-	tissue specimen	-
86.	+	-	-	-	tissue specimen	-
87.	+	+	-	-	tissue specimen	-

**Table 3:** Antifungal treatment (AFT), duration of AFT, surgical intervention, follow-up and infection outcome are presented. (*): death due to infection.

Case No.	AFT	Total Duration of AFT (Months)	Surgical Intervention	Follow-Up (Months)	Outcome
1.	Ketoconazole	-	+	38	Success
2.	Amphotericin B	-	+	50	Success
3.	Amphotericin B	-	+	22	Success
4.	Amphotericin B, Itraconazole, Ketoconazole	-	+	52	Success
5.	Amphotericin B	-	+	40	Failure *
6.	Fluconazole	-	+	40	Success
7.	Fluconazole	-	+	22	Success
8.	Amphotericin B, Fluconazole	-	-	62	Success
9.	Amphotericin B	-	+	38	Success
10.	Itraconazole, Fluconazole	12	+	12	Success
11.	Amphotericin B	-	-	-	Success
12.	Fluconazole	90	+	90	Success
13.	Fluconazole	36	+	36	Success
14.	Fluconazole	36	+	36	Success
15.	Fluconazole, Ketoconazole	12	+	12	Success
16.	Amphotericin B	20	+	20	Success
17.	Amphotericin B	6	+	6	Success
18.	Amphotericin B, Itraconazole	24	+	24	Success
19.	Fluconazole	6	+	6	Success
20.	Fluconazole	36	+	36	Success
21.	Amphotericin B	-	+	NR	Success
22.	Amphotericin B, Fluconazole	12	+	-	Success
23.	Amphotericin B, Fluconazole	9	-	60	Success
24.	Amphotericin B, Fluconazole	-	-	-	Unknown
25.	Fluconazole	30	+	30	Failure
26.	Fluconazole, Amphotericin B, Itraconazole, Caspofungin, Voriconazole	13	-	12	Success
27.	Fluconazole	14	+	14	Failure
28.	Fluconazole	-	+	-	Failure
29.	Fluconazole	1	+	48	Success
30.	Amphotericin B, Fluconazole	24	+	24	Success
31.	Itraconazole, Posaconazole	36	-	18	Success
32.	Fluconazole	6	-	18	Success
33.	Fluconazole	3	+	4	Success
34.	Amphotericin B, Fluconazole	-	+	-	Success
35.	Fluconazole	-	+	-	Success
36.	Amphotericin B, Fluconazole	-	+	-	Failure
37.	Amphotericin B, Itraconazole	18	+	-	Success
38.	Fluconazole, Amphotericin B	-	-	-	Failure
39.	Fluconazole, Amphotericin B	-	-	-	Success
40.	Fluconazole, Amphotericin B	-	-	-	Success
41.	Fluconazole, Itraconazole	-	-	-	Success
42.	Fluconazole, Amphotericin B	-	+	-	Success
43.	Fluconazole	-	+	-	Failure
44.	Fluconazole	-	+	-	Success
45.	Fluconazole, Amphotericin B, Voriconazole	-	+	-	Success
46.	Itraconazole	-	+	-	Success
47.	Itraconazole	-	+	-	Success
48.	Fluconazole, Amphotericin B	-	+	-	Success
49.	Fluconazole, Amphotericin B	-	+	-	Success
50.	Fluconazole, Amphotericin B	-	+	-	Success
51.	Amphotericin B	-	+	-	Success
52.	Fluconazole, Amphotericin B, Voriconazole, Caspofungin, Itraconazole	-	+	-	Success
53.	Fluconazole	-	+	-	Success
54.	Amphotericin B, Voriconazole, Itraconazole	-	+	-	Failure
55.	Itraconazole	-	+	-	Success
56.	Itraconazole, Amphotericin B	-	+	-	Success
57.	Fluconazole, Voriconazole	-	+	-	Success
58.	Fluconazole	-	-	-	Success
59.	Itraconazole, Voriconazole	-	-	-	Success
60.	Fluconazole, Amphotericin B	-	+	-	Success
61.	Itraconazole	-	+	-	Success
62.	Fluconazole	-	+	-	Success
63.	Amphotericin B	-	+	-	Success
64.	Fluconazole	-	-	-	Failure
65.	Fluconazole	-	-	-	Success
66.	Fluconazole, Itraconazole, Amphotericin B	-	+	-	Success
67.	Fluconazole	-	-	-	Success
68.	Fluconazole, Itraconazole, Amphotericin B	-	+	-	Success
69.	Fluconazole, Amphotericin B	-	+	-	Success
70.	Fluconazole	-	-	-	Failure
71.	Fluconazole, Amphotericin B	-	+	-	Success
72.	Fluconazole, Amphotericin B	-	+	-	Success
73.	Fluconazole, Itraconazole, Amphotericin B	-	-	-	Failure
74.	Fluconazole	-	+	-	Success
75.	Fluconazole, Amphotericin B	-	-	-	Success
76.	Fluconazole, Itraconazole, Voriconazole	-	+	-	Success
77.	Itraconazole, Amphotericin B	12	+	12	Success
78.	Itraconazole	6	+	NR	Success
79.	Fluconazole	12	+	1.5	Success
80.	Fluconazole	6	+	6	Success
81.	Amphotericin B	0.75	+	-	Failure *
82.	Fluconazole,	36	+	36	Failure
83.	Fluconazole	-	+	-	Success
84.	Amphotericin B.	-	+	24	Success
85.	Voriconazole, Amphotericin B	-	+	12	Failure
86.	Fluconazole	-	+	-	Unknown
87.	Fluconazole, Amphotericin B	-	+	-	Unknown

## Data Availability

Not applicable.
